# Switching from active vitamin D and phosphate supplementation to burosumab significantly corrects lower limb malalignment in pediatric X-linked hypophosphatemia

**DOI:** 10.1093/jbmr/zjaf079

**Published:** 2025-06-13

**Authors:** Leanne M Ward, Erik A Imel, David Frumberg, Lisa Dilworth, Catherine Siener, Zunqiu Chen, Stanley Krolczyk, Thomas O Carpenter, XLH Disease Monitoring Program Investigators, Andrea Arcari, Ambika Ashraf, Richard Baquero, Sanjukta Basak, Sasigarn Bowden, Oscar Brunetto, Thomas O Carpenter, Janet Crane, Kathryn Dahir, Bradley Dixon, Walter Guillermo Douthat, Pablo Florenzano, Ian Glass, Francis Glorieux, Gary Gottesman, Eric Gyuricsko, Ingrid Holm, Erik A Imel, Steven Ing, Suzanne Jan De Beur, Sarah Khan, Michael Levine, Regina Matsunaga Martin, Adriana Meza, Carolina Moreira, Neil Paloian, Anthony Portale, David Rodriguez-Buritica, Anna Ryabets-Lienhard, Jill Simmons, Puja Singh, Laila Tabatabai, Leanne M Ward, Halley Wasserman, Thomas Weber

**Affiliations:** Division of Endocrinology and Metabolism, Department of Pediatrics, Children’s Hospital of Eastern Ontario, University of Ottawa, Ottawa, ON K1H 8L1, Canada; Pediatric Endocrinology & Diabetology, Indiana University School of Medicine, Indianapolis, IN 46202, United States; Department of Orthopaedics and Rehabilitation, Yale University School of Medicine, New Haven, CT 06520, United States; Ultragenyx Pharmaceutical Inc., Novato, CA 94949, United States; Ultragenyx Pharmaceutical Inc., Novato, CA 94949, United States; Ultragenyx Pharmaceutical Inc., Novato, CA 94949, United States; Ultragenyx Pharmaceutical Inc., Novato, CA 94949, United States; Department of Orthopaedics and Rehabilitation, Yale University School of Medicine, New Haven, CT 06520, United States

**Keywords:** X-linked hypophosphatemia, lower limb malalignment, burosumab, genu varum, genu valgum, active vitamin D, phosphate

## Abstract

X-linked hypophosphatemia (XLH) is a rare disorder of renal phosphate wasting and dysregulated active vitamin D metabolism, ultimately presenting as rickets and osteomalacia, among other manifestations. Lower extremity deformity (genu valgum and/or varum) is frequent in this pediatric population. Despite prompt active vitamin D and phosphate supplementation (active D/Pi), many patients require corrective surgery for lower limb malformation. Burosumab has demonstrated improvements in lower limb malalignment in children with XLH in several studies. We expand on those reports by assessing mechanical femoral tibial angle (mFTA) change in patients enrolled in the XLH Disease Monitoring Program (DMP), (NCT03651505) to determine the impact of initiating burosumab treatment after a history of active D/Pi. Included patients had either switched from active D/Pi to burosumab treatment at the discretion of their treating physician or as part of a burosumab clinical trial, or remained on active D/Pi through Year 3 of the DMP. Year 3 radiographs were compared with baseline to assess mFTA change and gauge improvement. Additional multivariate factor analysis examined 24 attributes to determine which had the greatest association with mFTA change. Change in mFTA was assessed for each limb independently. A greater proportion of limbs of patients switching from active D/Pi to burosumab had improved mFTA compared with those remaining on active D/Pi (*p* < .023). Odds ratios comparing limbs that improved to those that did not showed that switching to burosumab yields a significantly greater chance of improvement than continuing active D/Pi (OR [95% CI]: 4.38 [1.09-17.50]; *p* = .0469). Factor analysis identified younger age at burosumab initiation (*p* = .001) and lower baseline height Z-score (*p* = .006) as being significantly associated with greater change in mFTA Z-score. This study shows that switching to burosumab significantly improves lower limb malalignment in children with XLH over benefits conferred by active D/Pi, with early burosumab initiation providing the greatest benefit.

## Introduction

X-linked hypophosphatemia (XLH) is a rare, progressive, hereditary disorder of renal phosphate wasting, which has an incidence of approximately 1:20 000.^1^^,^^2^ Underlying loss-of-function pathogenic variants in the phosphate-regulating endopeptidase homolog X-linked (*PHEX*) gene lead to excess fibroblast growth factor 23 (FGF23), which in turn cause reduced expression of sodium-phosphate cotransporters (renal phosphate wasting) and dysregulated active vitamin D metabolism (decreased synthesis and increased catabolism).^3^^,^^4^ In children with XLH, these biochemical alterations typically occur in concert with rickets and osteomalacia,^2^ as well as short stature, bone pain, dental abscesses, craniosynostosis, and muscle weakness. Lower extremity deformity (genu valgum and/or varum) is also a frequent manifestation of the pediatric XLH phenotype, typically manifesting in early childhood around the time of weight-bearing.^4^^,^^5^ Recent studies have shown that genu varum is reported in 45%-64% of children with XLH, and genu valgum in 7%-35%.^1^^,^^6^^,^^7^

As a progressive disorder, appropriate disease management early in life is essential to minimize the potential lifelong disease burden associated with XLH.^7–9^ Bearing weight on malaligned and poorly-mineralized lower extremities is associated with osteomalacic fractures,^10^ progressive lower limb deformity,^11^ and osteoarthritis^10^ in adulthood, while early treatment improves lower limb deformity,^12–14^ along with growth velocity, final height,^15^ and dental mineralization.^9^^,^^16^

Historically, therapy for XLH consisted of active vitamin D supplementation combined with multiple daily doses of oral phosphate (active D/Pi) to improve skeletal mineralization^4^^,^^17^^,^^18^; however, even with prompt and adequate treatment (<1 yr of age), this often fails to fully correct skeletal malalignment and short stature.^2^ Reports suggest that approximately 40% of patients with XLH who receive prompt active D/Pi with close monitoring still require later surgery to correct lower limb deformities, improve gait, ameliorate leg length discrepancies, or correct malalignment.^2^ The risk of hypercalcemia, hypercalciuria, hyperparathyroidism, and nephrocalcinosis with active D/Pi as well as gastrointestinal intolerance to oral phosphate administration limit adherence to adequate dosing strategies of these medications.^2^^,^^4^^,^^18–20^

Active D/Pi does not fully address rickets and osteomalacia in XLH and their related complications, particularly lower limb malalignment. Therefore, surgical intervention may be required to correct such deformities.^19^^,^^21^ Up to 2/3 of children/adolescents with XLH have been reported to undergo lower limb surgery, most commonly hemiepiphysiodesis (guided growth treatment) in milder cases of varus and valgus deformities.^1^^,^^2^^,^^18^^,^^22^^,^^23^ In more severe cases or those unresponsive to treatment, persistent and progressive malalignment is managed with osteotomy post-epiphyseal fusion^1^^,^^23^^,^^24^; however, deformity has been reported to recur in up to 90% of patients within 5-12 mo following initial surgery, and in some cases, 2-3 operations are required throughout growth.^23–25^ Furthermore, the inherent risks of surgery in this setting have been noted. In a 2022 systematic review, Paludan et al. described a mean incidence of 1 complication per surgery (tibial/femoral epiphysiodesis, tibial/fibular/femoral osteotomy, and hardware removal surgery), and with significant severity, including recurrent or residual deformity, abdominal pain, stress fracture, sensory nerve damage, hardware infection or protrusion, muscle fatigue, knee locking, waddling gait, irritation, tight Achilles tendon, keloid scar, and reduced knee extensor power.^26^ Moreover, in 28% of surgeries, these complications were such that the planned treatment goal could not be achieved, with half of these cases resulting in permanent sequelae or new pathology.

The approval of burosumab, a fully human monoclonal antibody to FGF23, provides a more pathophysiology-targeted treatment option for patients with XLH, with proven efficacy in children compared to active D/Pi.^27^^,^^28^ Several studies have described improvements in lower limb malalignment in children with XLH. In a recent analysis of 116 limbs of children with XLH who participated in clinical trials, treatment with burosumab was capable of correcting the mechanical femoral tibial angle (mFTA) of varus and valgus lower limbs to a neutral alignment without surgical intervention.^29^ In the 2 clinical trials assessed, the proportion of children with normal alignment at baseline was only 19.2%, which improved to 58.3% over 160 wk of burosumab.^29^ Other analyses from the same trials have similarly described significant improvements in lower limb deformity using Radiographic Global Impression of Change (RGI-C) scores in children treated with burosumab compared with those on active D/Pi.^13^^,^^28^^,^^30^

The present analysis provides a more detailed assessment of the longer-term impact of burosumab in children with XLH previously-treated with active D/Pi on lower limb malalignment in children with XLH by expanding the cohort from the above 2 trials and examining the larger patient population enrolled in the prospective XLH Disease Monitoring Program (DMP); (NCT03651505). In addition, we use a novel approach to quantify lower limb malalignment, by converting femoral tibial angle raw scores to Z-scores, allowing for direct comparison of results to healthy age-matched norms.

## Materials and methods

This post-hoc analysis included children <18 yr of age with XLH enrolled in the DMP as of March 2023 with no history of lower limb malalignment surgery. Subjects enrolled in the XLH DMP included those who had previously (ie, pre-DMP baseline) switched from active D/Pi to burosumab as part of a previous clinical trial (CL201 [NCT02163577], CL205 [NCT02750618], CL301 [NCT02915705]), those who initiated burosumab pre- or post-DMP baseline via commercial access at the discretion of their treating physician, and those who remained burosumab-naïve at baseline DMP and throughout the three years of DMP observation (but still received active D/Pi).^13^^,^^14^^,^^31^ Subjects were excluded if they had undergone lower limb corrective surgery before or after their baseline visit during the period of follow-up. A detailed patient disposition diagram is included in the Supplementary Materials.

The XLH DMP study protocol is a fully-sponsored study compliant with the International Council for Harmonization of Technical Requirements for Pharmaceuticals for Human Use Good Clinical Practice. The XLH DMP was approved by the appropriate independent ethics committee or institutional review board for each center and complied with the ethical principles of the Declaration of Helsinki and Good Clinical Practice. Parents or guardians provided written informed consent for study participation. Children and adolescents gave written consent or assent per local guidelines.

Standing, full-length leg radiographs were assessed to determine the impact of switching from active D/Pi to burosumab treatment on lower leg malalignment through measurement of the mFTA, a standard measurement of lower limb angular (genu varum and valgum) deformity.^11^^,^^29^ As the present study is a retrospective analysis of historical data, variability exists in the timing of baseline measures.

Baseline radiographs were obtained at the earliest available timepoint for each patient (*n* = 136), referred to as “Baseline” throughout, with additional radiographs scheduled for collection at DMP Years 1, 3, 5, 7, and 10. For those who had participated in a prior burosumab clinical trial before entering the DMP (with prior trials including CL201 [NCT02163577], CL205 [NCT02750618], CL301 [NCT02915705]), baseline was considered the screening visit of the prior clinical trial (*n* = 45) before initiating burosumab (median 29 d [Q1, Q3: 22, 56] between the baseline radiograph and burosumab initiation; range: 7 to 522 d). For those patients who first received burosumab commercially (*n* = 82), baseline was considered the time of enrollment in the XLH DMP. In some of these patients (44/82; 54%), burosumab initiation occurred 84 (1, 232; range: 0 to 940) days following the collection of baseline radiographs. The remaining 38/82 patients (46%) initiated burosumab treatment 12 (5, 44; range: 2 to 310) days before baseline imaging. Finally, a subset of patients in this study never received burosumab, instead remaining on active D/Pi. Baseline for these patients was considered DMP baseline. All burosumab-treated subjects received burosumab for a minimum of 3 yr at the time of analysis; available data through DMP Year 3 are reported herein.

**Figure 1:**
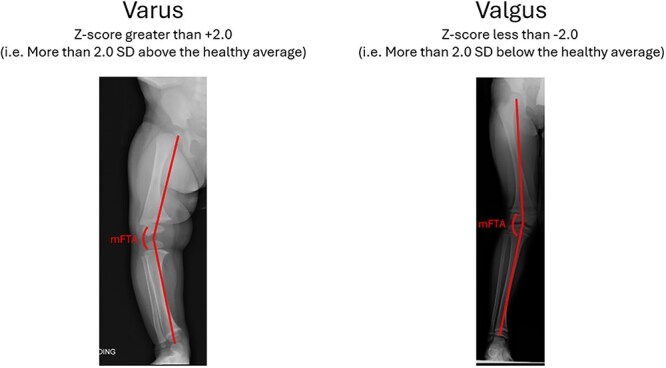
Mechanical femoral tibial angle (mFTA) measurement. mFTA measurements were performed at each time point (baseline and DMP Year 3) by a single reader masked to the subject identification number and treatment group. The reader reported the mFTA of each limb independently, measuring from the center of the femoral head to the midpoint of the knee joint, and from the midpoint of the knee joint to the midpoint of the ankle joint. mFTA Z-scores greater than +2.0 (ie, more than 2.0 SD above the healthy average) were considered varus (see left), while those less than −2.0 (ie, more than 2.0 SD below the healthy average) were considered valgus (see right).

Radiographs were acquired according to protocol specifications: bilateral, anterior-posterior views in the upright (standing) position, with single images containing both legs from above the iliac crests to below the ankle. Subject preparation and positioning were performed per the site’s institutional standard of care. Image quality standards were established to ensure quality control. Images were submitted to an independent central reading vendor (Clario) and graded at each time point (baseline and DMP Year 3) by a single reader who was masked to the subject study identification number and treatment category. The reader reported the mFTA of each limb independently, measuring from the center of the femoral head to the midpoint of the knee joint, and from the midpoint of the knee joint to the midpoint of the ankle joint (Figure 1). Both baseline and Year 3 measurements were evaluated by the central reader at the same time. mFTA raw results were transformed to age-matched Z-scores for analysis.^32^

Normal and abnormal lower limb alignment were defined as follows^32^:

1. Normal lower limb alignment: mFTA Z-score within 2.0 SD of the healthy average (ie, ≥−2 to ≤+2).
2. Genu varum: Z-score greater than +2.0 (2.0 SD above the healthy average).
3. Genu valgum: Z-score less than −2.0 (2.0 SD below the healthy average.

**Figure 2:**
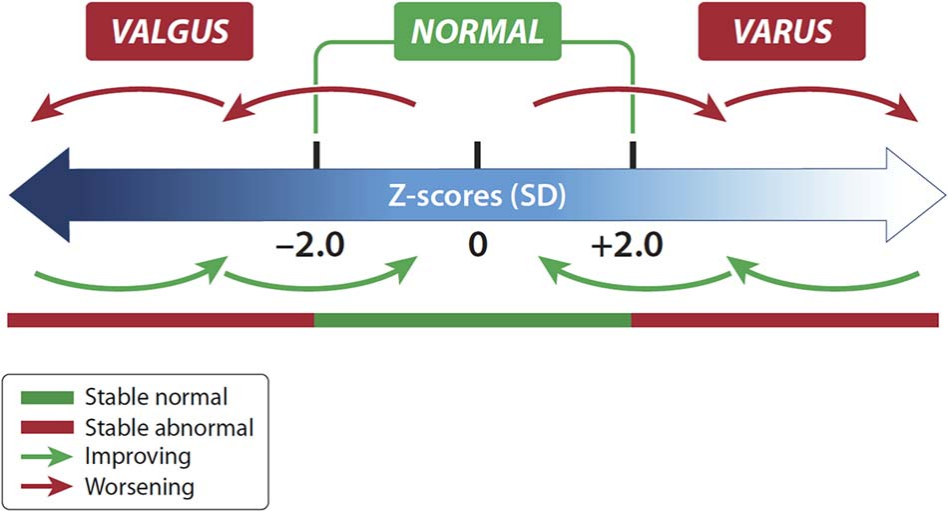
Definitions of normal and change from baseline. mFTA Z-score changes from baseline at DMP year 3 were categorized as stable (normal/abnormal), improving, or worsening based on initial and final measures. Any baseline score of “normal” (ie, within 2 SD of the healthy average) with a corresponding final value outside of the normal range, or a baseline score outside of the normal range deviating further from the normal range by at least 1.0 SD at final measure, was considered “worsening.” Any baseline score of “abnormal” (ie, beyond 2.0 SD of the healthy average in either direction) with a corresponding final value within the normal range, or with a final value at least 1.0 SD improvement towards the normal average, was considered “improving.” Scores that remained within the normal range of ±2.0 SD of the age-matched healthy average at baseline and follow-up were considered stable normal; those that changed less than 1.0 SD in the presence of both baseline and follow-up values that were outside of the normal range were considered stable abnormal.

The differences from DMP Year 3 and baseline were characterized as follows (with definitions pictorialized in Figure 2):

1. Improving:
  - Improvement to normalization = change from outside to within 2.0 SD of the age-matched healthy average (with ±2 SD of the healthy average representing “the normal range”).
  - Improvement with residual malalignment = greater than 1.0 SD change towards the healthy average without crossing into ±2.0 SD of the healthy average.
2. Worsening:
  - Change from within to outside of 2.0 SD of the healthy average.
  - Progression from outside the normal range to an even greater departure from normal by at least 1.0 SD, or from abnormal varus to abnormal valgus and vice versa.
3. Stable:
  - Remaining within the normal range of ±2.0 SD of the age-matched healthy average (stable normal).
  - A change less than 1.0 SD when both the initial (ie, baseline) and follow-up values were outside of the normal range (stable abnormal).^32^

Note that 1.0 SD was selected as the minimum divergence as this equates to an approximately 2° change from baseline, which has been described previously as the clinically meaningful difference of angular measures.^32^^,^^33^

Assessment of mFTA change from baseline at DMP Year 3 was used to determine the proportion of subjects on burosumab who demonstrated improvement relative to their pre-treatment mFTA.

A linear mixed-effects model examined factors associated with greater improvements from baseline over 3 yr in mFTA Z-score. This analysis explored 24 possible factors spanning patient-reported outcomes, dosing, laboratory outcomes, disease state, and patient characteristics. The full list of factors examined is included in the Supplementary Methods.

### Statistical methodology

Descriptive summary statistics for baseline demographic information and key laboratory results were presented using means, SDs, and proportions, as appropriate. Fisher’s exact test was performed between subjects who received burosumab and those on active D/Pi to assess the impact of treatment on mFTA improvement, worsening, or stabilization. A linear mixed-effects model for repeated measures was employed to assess the factors contributing most to the variability in mFTA. This model accounted for intra-subject correlations arising from the left and right leg measurements taken from the same subjects. Stepwise variable selection was conducted to identify the most significant predictors influencing mFTA variability. Odds ratio calculations were employed to compare the likelihood of improvement or stabilization of lower limb malalignment in subjects receiving burosumab vs those on active D/Pi. All statistical analyses were performed using SAS version 9.4 and R version 4.4.1.

## Results

This analysis included 136 subjects from the XLH DMP (127 who switched from active D/Pi to burosumab; 9 remained on active D/Pi with no burosumab exposure) (Table 1). Of these, 45 had initiated burosumab as part of a previous clinical trial (median [range] burosumab duration at Year 3 of the DMP: 72 [40.3100.7] mo), 82 were burosumab-naïve at DMP baseline, but initiated burosumab through commercial availability at the discretion of their treating physician (36.2 [1.4, 54.8] mo), and 9 were burosumab-naïve at DMP baseline and remained so during the 3 yr of observation reported herein. All burosumab-treated subjects had previously received active D/Pi for XLH consisting of phosphate and/or active vitamin D supplementation. At baseline, subjects who went on to receive burosumab had received prior active D/Pi for 5.3 (3.6) yr, while those remaining on active D/Pi had received their therapy for 4.6 (3.2) yr. By DMP Year 3, 127/136 subjects (93%) had received burosumab for 4.0 ± 1.8 yr, including exposure during prior clinical trials, while 9/136 (7%) remained on active D/Pi for the duration. Laboratory values at final visit are included in Supplementary Table. All 127 burosumab-treated subjects received burosumab for at least 3 yr.

**Table 1:** Baseline characteristics. Baseline characteristics of patients included in the analysis are reported based on treatment group (those who switched to burosumab and those who remained on active D/Pi) and then summed.

**Characteristic**	**Active D/Pi to burosumab-treated**	**Active D/Pi only** ^**a**^	**Total**
**Subjects, *n***	127	9	136
**Age, yr; mean (SD)**	7.3 (3.9)	6.4 (3.0)	7.2 (3.9)
**Sex, Female; *n* (%)**	70 (55.1%)	4 (44.4%)	74 (54.4%)
**Duration of prior active D/Pi exposure, yr; mean (SD)**	5.3 (3.6)	4.6 (3.2)	5.2 (3.6)
**BMI; mean (SD)**	18.8 (4.1)	18.5 (2.8)	18.7 (4.1)
**Height Z-score; mean (SD)**	−1.6 (1.4)	−1.5 (0.9)	−1.6 (1.4)
**Serum phosphate, mmol/L; mean (SD)**	1.0 (0.2)	0.9 (0.1)	1.0 (0.2)
**Serum phosphate Z-score** ^**34**^ **^,^** ^**35**^ **; mean (SD)**	−2.6 (1.0)	−3.2 (0.7)	−2.6 (1.0)
**Alkaline phosphatase, U/L; mean (SD)**	403 (150)	447 (161)	405 (150)
**Serum alkaline phosphatase Z-score; mean (SD)**	2.4 (1.9)	2.8 (2.6)	2.4 (2.0)

a Active D/Pi-only patients were considered “burosumab-naïve.”

Abbreviation: BMI, body mass index.

**Figure 3:**
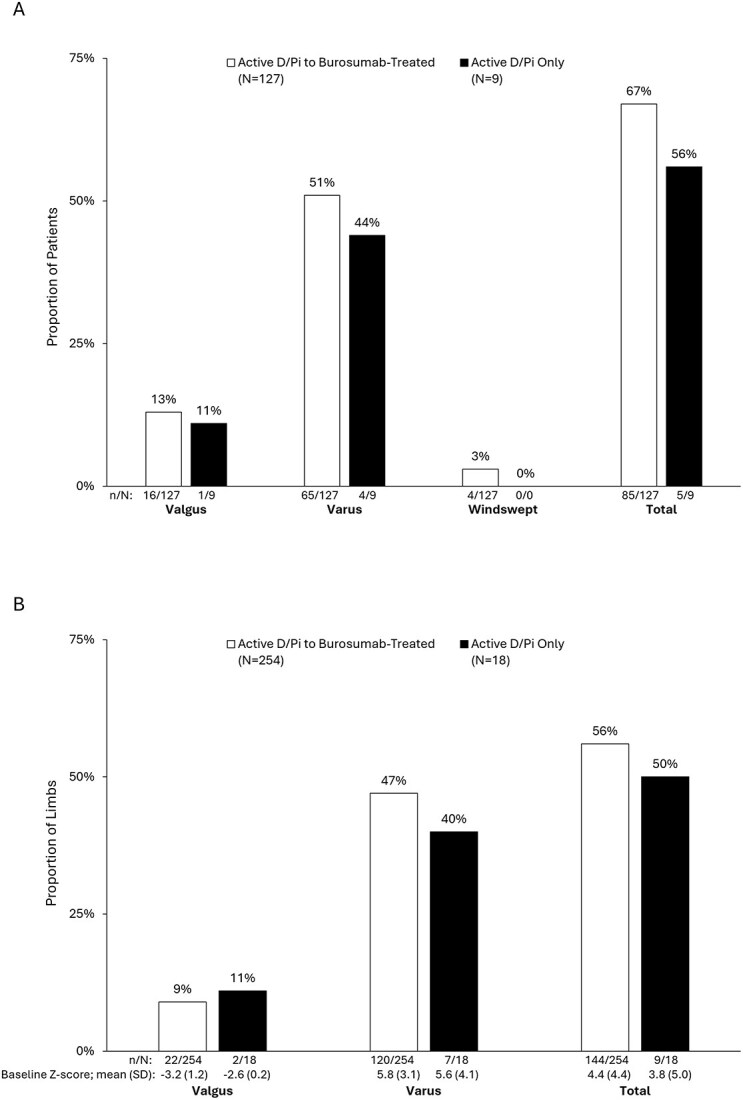
Proportion of patients (A) and limbs (B) with lower limb malalignment at baseline and the corresponding mean Z-scores for Varus and valgus deformities. mFTA Z-scores were categorized by baseline deformity (valgus, varus, or one of each [windswept]) at the patient (A) and limb (B) levels grouped by treatment.

Of the 127 children who received burosumab treatment, 85 (67%) had at least one leg in varus or valgus at baseline, compared with 5/9 (56%) of those on active D/Pi (Figure 3). Across treatments, children with varus or valgus deformity at baseline were, on average, younger (5.8 ± 3.2 yr) and shorter (height Z-score −1.8 ± 1.4) than those without deformity (8.1 ± 3.8 yr, and −0.91 ± 1.1), both *p* < .001 via Wilcoxon rank sum test. Of the 254 legs of children who received burosumab, 142/254 (56%) had lower limb malalignment at baseline comparable to the 9/18 (50.0%) of children who remained on active D/Pi. Notably, the severity of baseline valgus and varus deformities was numerically greater in limbs of children that went on to receive burosumab at baseline (using radiographs pre-burosumab initiation) compared with those that remained on active D/Pi through DMP Year 3; however, statistical analysis was not conducted due to small sample sizes.

**Figure 4:**
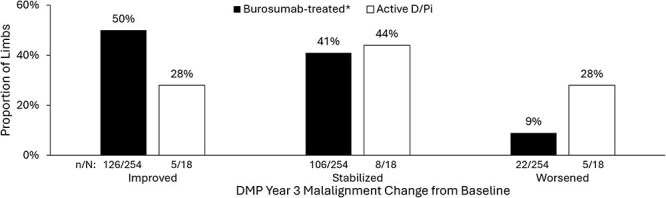
mFTA change from baseline at DMP Year 3. The change in mFTA Z-score from baseline at DMP Year 3 was used to group limbs as improved, stabilized, or worsened within each treatment group. The proportion of limbs in each grouping is reported. Fisher’s exact test comparing the proportions of patients within each treatment group that improved, with those that worsened, yielded a *p*-value of .023. *Mean burosumab duration 4.0 ± 1.8 yr, including exposure during prior clinical trials.

**Figure 5:**
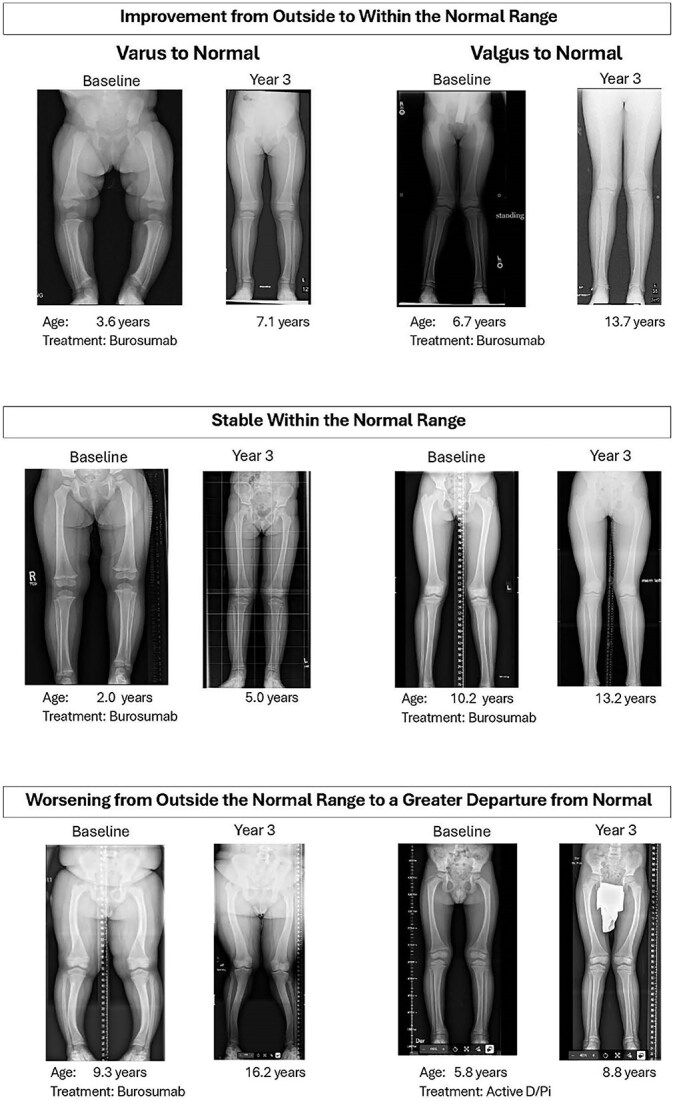
Radiographic images of mFTA outcomes. Standing, full-length lower extremity radiographs were obtained at baseline and DMP Year 3 according to protocol specifications: bilateral, anterior-posterior views in the upright (standing) position, with single images containing both legs from above the iliac crests to below the ankle. Subject preparation and positioning were performed per the site’s institutional standard of care. Image quality standards were established to ensure quality control. Representative images of improvement (top), stabilization (middle), and worsening (bottom) are provided.

At DMP Year 3, a greater proportion of limbs receiving burosumab improved their mFTA (126/254, 50%) from baseline vs those receiving active D/Pi (5/18, 28%) (Figure 4). Moreover, a smaller proportion of limbs on burosumab (22/254, 9%) worsened compared with those on active D/Pi (5/18, 28%) over the same period. Fisher’s exact test comparing the proportions of limbs improving with those worsening across treatment groups yielded a *p*-value of .023. Switching to burosumab treatment from active D/Pi clinically improved lower limb malalignment (mean absolute magnitude of Z-score change from baseline: 4.1) compared with remaining on active D/Pi (mean absolute magnitude of Z-score change from baseline: 1.5) by DMP Year 3. The mean absolute Z-score change in those whose mFTA stabilized was 0.1 vs −0.1 in the burosumab-treated vs active D/Pi groups, respectively. Finally, the mean absolute Z-score change in those whose mFTA worsened was −2.0 for the burosumab-treated children vs −2.3 for the active D/Pi-treated children, respectively. Notably, the changes from baseline achieved by the burosumab-treated group were more favorable than those experienced by the active D/Pi treatment group. Radiographic images depicting improvement, stabilization, and worsening (according to pre-specified definitions) are included in Figure 5.

The odds ratios comparing limbs with baseline malalignment that improved to those that did not improve (stabilized/worsened) indicated that switching to burosumab yielded a significantly greater odds of improvement than continuing active D/Pi (odds ratio [95% CI] of 4.38 [1.09-17.50]; Fisher’s exact test: *p* = .0469). Similarly, for limbs with no malalignment at baseline, those on burosumab had a significantly greater odds of stabilization vs worsening compared with those on active D/Pi (4.80 [1.17-19.80]; Fisher’s exact test: *p* = .0402).

**Figure 6:**
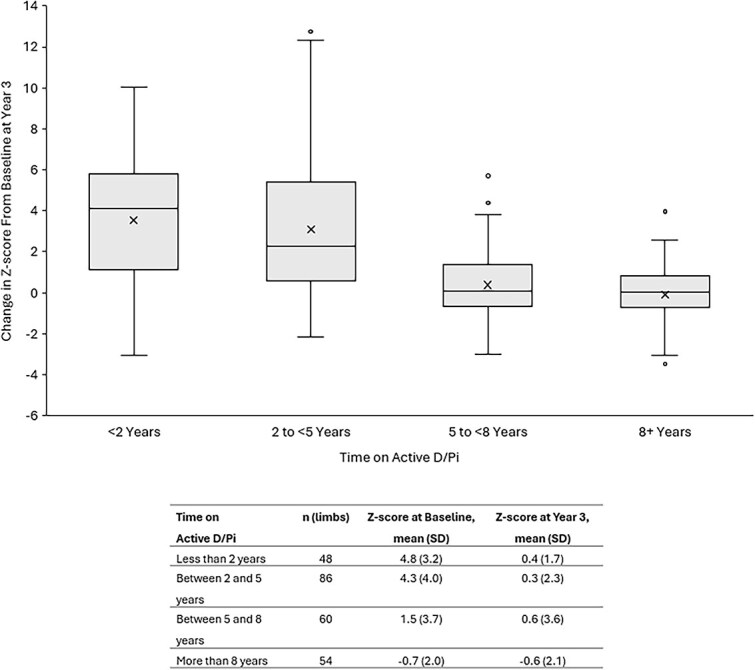
Mean change in mFTA Z-score by time on active D/Pi between baseline and Year 3. Mean change in mFTA Z-score is presented with limbs grouped by duration of prior active D/Pi treatment. Here, the vertical limits of the box represent quartiles 1 and 3, the whiskers represent the minimum/maximum values, the inner horizontal line represents the median, the “X” represents the mean, and the circles represent outliers. Mean (SD) mFTA Z-scores at baseline and Year 3 are reported below (table).

Assessment of the impact of duration of active D/Pi was conducted to better understand the consequences of delaying burosumab treatment (Figure 6). This analysis included the duration of active D/Pi in those who went on to receive burosumab, and the results of the baseline and Year 3 lower limb alignment assessments for the corresponding 248 limbs Due to the small sample size, the absolute change in lower limb malalignment is reported without indicating the direction of change. This outcome is calculated as the absolute value of the baseline Z-score minus the absolute value of the Year 3 Z-score (ie, without the assigned positive or negative directionality, but rather, the magnitude of the overall change). Here, delaying transition to burosumab from active D/Pi was associated with a lesser mean improvement of mean mFTA Z-score.

Univariate analysis examined key factors associated with correction including time on active D/Pi and baseline height Z-score. Analysis of mFTA Z-score at baseline with height Z-score identified a significant negative relationship (correlation coefficient: −0.47), confirming that limbs of shorter patients exhibited more severe deformity at baseline (*p* < .0001). Based on these findings, a broader subsequent multivariate factor analysis assessed 24 possible variables including prior treatment, baseline height Z-score, and age (Supplementary Methods) to determine their association with the change in mFTA from baseline at DMP Year 3. Of these 24 factors, younger age at first burosumab dose (*p* = .001) and lower height Z-score at baseline (*p* = .006) were significantly associated with greater change in mFTA Z-score. To assess the direction of change from baseline, all limbs with baseline varus (Z-score > +2.0) or valgus (Z-score < −2.0) deformity were examined for both those who switched to burosumab and those who remained on active D/Pi. Among the 127 limbs with varus deformity at baseline, subjects that switched to burosumab (*n* = 120) demonstrated an improvement in mean mFTA Z-score of −4.0 (3.0), indicating a substantial overall reduction in varus deformity. However, those remaining on active D/Pi (*n* = 7) improved the mean mFTA Z-score by −1.8 (1.1), reflecting a lesser reduction in varus deformity. For the 24 limbs with valgus deformity at baseline, the burosumab-treated group (*n* = 22) improved the mean Z-score by +1.5 (1.4), indicating a reduction in valgus deformity, while the active D/Pi group (*n* = 2) exhibited a mean (SD) change of +0.04 (3.6), suggesting minimal improvement. No statistical comparisons were performed for these continuous outcomes due to the very small sample size of the active D/Pi group (*n* = 2).

At DMP Year 3, 40/142 of limbs (28%) on burosumab that had mFTA malalignment at baseline were left with residual malalignment (Table 2). Of the 22 limbs with abnormal valgus at baseline, 8 (36%) remained abnormal at Year 3. Similarly, of the 120 limbs with abnormal varus at baseline, 27 (23%) remained abnormal at Year 3. Five of the 120 (4%) limbs with abnormal varus at baseline crossed over to abnormal valgus at Year 3. Here, the limbs with varus deformities at both timepoints improved by a mean Z-score of −3.6 (3.6) at DMP Year 3, while the limbs with valgus deformities at both timepoints improved by +0.6 (0.9). In the 5 limbs with baseline varus deformities observed in 4 subjects that crossed over to abnormal valgus deformity at Year 3 Z-score changed from 2.7 (0.1) to −4.1 (1.2) over that time frame, thus an overall change in mean Z-score of 6.8 (1.1) was observed.

**Table 2:** Baseline follow-up Z-score changes of patients who switched to Burosumab prior to baseline, or who switched to burosumab between baseline and Year 3. The proportions of limbs with baseline malalignment improving to normalization (top) and those with residual malalignment (bottom) at Year 3 are reported, further grouped by baseline deformity (valgus or varus). For each, the mean (SD) baseline, Year 3, and change in mFTA Z-scores are presented.

	**Baseline deformity**	
	**Valgus**	**Varus**
**Baseline Malalignment Improved to Normalization at Year 3**		
** Proportion of limbs improving to normalization at Year 3**	14/22 (64%)	88/120 (73%)
** Z-score at baseline, mean (SD)**	−3.2 (1.2)	+5.2 (2.4)
** Z-score at Year 3, mean (SD)**	−0.5 (1.2)	+0.02 (1.0)
** Z-score change from baseline at Year 3, mean (SD)**	+2.7 (1.8)	−5.2 (2.5)
**Baseline malalignment with residual malalignment at Year 3**		
** Proportion of limbs with residual malalignment at Year 3**	8/22 (36%)	27/120 (23%)
** Z-score at baseline, mean (SD)**	−3.3 (1.1)	+8.3 (3.9)
** Z-score at Year 3, mean (SD)**	−2.6 (0.5)	+4.7 (3.2)
** Z-score change from baseline at Year 3, mean (SD)**	+0.6 (0.9)	−3.6 (3.6)

## Discussion

We examined data from children enrolled in the XLH DMP to determine the impact of treatment on mFTA malalignment. Subjects who had received at least 3 yr of treatment with burosumab following previous treatment with active D/Pi were compared with those who received only active D/Pi. At Year 3 of the DMP, we observed significant greater improvement from baseline in mFTA in subjects who had switched from active D/Pi to burosumab as compared to those remaining on active D/Pi (*p* = .023). Furthermore, the mFTA of a greater proportion of limbs from children on burosumab improved and a smaller proportion worsened, as compared to active D/Pi. This finding was further supported by significantly higher odds ratio for maintaining normal baseline alignment and of improving baseline malalignment with burosumab compared with active D/Pi. We propose that the improved renal phosphate reabsorption and vitamin D synthesis associated with burosumab-mediated inhibition of FGF23 results in better healing of rickets and osteomalacia, which are fundamental for correcting lower limb malalignment. We note that the improved renal phosphate handling that occurs in response to FGF23 neutralization is well-recognized; however, also contributing to the improved serum phosphate levels is the augmentation of renal 1-alpha-hydroxylase activity arising from FGF23 neutralizing therapy, as shown in the pediatric (and adult) clinical trials.^13^^,^^28^ This enhances 1,25(OH)_2_D synthesis, which in turns stimulates intestinal phosphate absorption. In addition, factor analysis showed that the most impactful variables associated with change in mFTA were young age at burosumab initiation and lower height Z-score at baseline. Analysis of duration of active D/Pi suggested a strong correlation of longer exposures with smaller changes in mFTA. Finally, among limbs with residual mFTA deformity despite at least 3 yr of burosumab treatment, improvement from pre-treatment alignment was observed in 7/40 limbs (18%), and evident in the presence of either baseline varus and valgus deformities.

To our knowledge, this is the first study to demonstrate the relationship between age of treatment initiation and the potential for improvement in lower limb malalignment with burosumab. We speculate that the more rapid growth occurring in young childhood may allow for more dynamic change in a less mature growth plate and thus allow for greater correction of deformity. Furthermore, the accompanying greater residual growth potential in a younger patient may also influence the impact of burosumab on lower limb malalignment, since skeletal modeling is a growth-dependent process. In addition, it stands to reason that a rickets therapy is more likely to reduce the duration of exposure to loading of the skeleton with an aberrant mechanical axis, thereby potentially reducing the adverse biomechanical effects of malalignment deformity on the growth plates. Overall, the goal of early treatment is to promote skeletal mineralization, improve linear growth through endochondral bone formation, correct leg deformities, and obviate the need for surgical intervention.^15^^,^^19^ This belief is supported by the strong correlation between length of active D/Pi exposure and lack of improvement in malalignment observed in this study. We also report the significant relationship between shorter stature at baseline and greater degree of lower limb malalignment. This suggests there may be greater room for improvement in overall patient height with burosumab treatment in those with the most significant lower limb deformity. As a minimum, our data support that short stature is not necessarily disadvantageous for potential improvement in the lower limb malalignment of XLH.

A considerable proportion of those with lower limb deformity at baseline in the burosumab-treated group improved to normal limb alignment by Year 3 of the DMP (102/142; 71.8%), while 40/142 (28.2%) of this group still had residual malalignment. However, the latter group had more extreme baseline deformity (Z-score: −3.3 [valgus] and +8.3 [varus] than those with complete resolution of malalignment (−3.2 [valgus] and + 5.2 [varus]). Nevertheless, this group improved toward normality considerably (Z-score change from baseline of +0.6 [valgus] and −3.6 [varus]). Of note, valgus deformity was associated with a lesser degree of improvement as compared to varus deformity (in those with residual malalignment); however, this equated to a similar proportion of patients achieving normality with valgus (64%) or varus (73%) malalignment at baseline.

As the 10-yr DMP study continues, these subjects will be followed to determine the ongoing trajectory of alignment. Five limbs from four patients with residual malalignment crossed over from varus to valgus deformity by Year 3. Additional follow-up analyses will investigate specific factors associated with limited improvement despite treatment; indeed, the baseline BMI of 1 subject with both limbs in the varus-valgus crossover group was 44.1, suggesting that obesity may be an impediment to alignment correction in XLH, despite treatment. This line of thinking has been supported by studies showing that idiopathic genu valgum is associated with obesity in children and adolescents.^36^^,^^37^

The findings reported herein are consistent with previous studies that have suggested improvements in the RGI-C lower limb deformity score^13^^,^^28^^,^^30^ and specifically in mFTA.^29^ In the latter, Frumberg et al. examined improvements in mFTA in patients from 2 burosumab clinical trials (CL205 [NCT02750618], CL301 [NCT02915705]), reporting a large proportion of subjects with XLH achieving normal mFTA alignment following 64 and 160 wk of burosumab treatment (from 19.6% of children at baseline to 37% at 64 wk and to 58.3% at 160 wk).^29^ In the present analysis, 71.8% of subjects with deformity at baseline improved to normality by DMP Year 3 during burosumab treatment. Notable differences between the current study and that of Frumberg et al. include a larger sample size in the present study, a longer follow-up, inclusion of a real-world population along with trial subjects, and a novel methodology to express mFTA - age-matched Z-scores (which facilitated the analysis and interpretation of the data in a pediatric population, an important strategy given that even healthy children transition from on average 15° of genu varum at birth to a slight genu valgum (about 5°) by 6 yr of age. In addition, there are sex-related differences in lower extremity alignment that need to be taken into consideration, with boys having higher mFTA than girls.^38^

Our observations are critical to the treatment paradigm of children with XLH, as they support the growing body of evidence that burosumab treatment improves lower limb malalignment which, in turn, may obviate the need for corrective surgery, particularly in those who initiate treatment early. Angular deformity in children contributes to considerable disease burden in adulthood if left uncorrected.^7–9^ Adverse long-term outcomes include the need for osteotomy surgery, gait abnormalities, the need for mobility devices, leg length discrepancies, pain, fractures, osteoarthritis due to improper alignment, and ongoing pain due to worsening of existing malalignment.^39–42^ Moreover, it has been proposed that early correction of lower limb malalignment can decrease the risk of osteoarthritis in adulthood.^43^ Active D/Pi often fails to address these consequences of XLH, leading to approximately 40% of patients requiring surgery, even with prompt active D/Pi initiation in children less than 1 yr of age.^2^^,^^15^ In the present analysis, 272 limbs had a mean exposure duration of 5.2 yr of active D/Pi at baseline; despite this, 151/272 (56%) limbs had lower limb malalignment. After switching to burosumab therapy, 126/142 limbs (89%) with baseline malalignment showed marked improvement without surgical intervention. Furthermore, these findings were most highly associated with younger age at burosumab treatment initiation and shorter stature, underscoring the importance of early XLH identification and treatment, and the fact that short stature at a young age does not portend a less favorable outcome where lower limb malalignment is concerned. Together, these observations support the shift in the XLH treatment paradigm towards burosumab as the new convention and early initiation of treatment.

The present study is limited by the variable burosumab treatment duration, stemming from differences in the timing of treatment initiation and subsequent time-on-treatment. Despite this, our study provides data on the longest duration of burosumab exposure in relationship to lower limb alignment to date (mean: 4.0 yr). Indeed, some subjects received active D/Pi for a period between baseline and burosumab initiation; however, this would suggest that the benefits of switching to burosumab presented herein are an underestimation, further supporting the change from active D/Pi. Further, the present study shows the evolution of lower limb malalignment on burosumab and active D/Pi in the real-world setting, using a quantification technique (mFTA) that is part of routine clinical care for pediatric orthopedic surgeons and using a unit of measure (Z-score) with which pediatric health care professionals can identify. Another limitation of this study is that the radiographic approach assessed only coronal plane malalignment and did not permit evaluation of torsional deformity, which can also influence a child’s gait and overall mobility status^7^; this aspect of lower limb alignment requires further study of sagittal and axial plane alignment over the longer-term. Further, this study excluded persons having corrective surgery before (*n* = 7) or after (*n* = 16) baseline, which may have resulted in overestimating treatment effect. Of note about 11% of the subjects treated with burosumab were excluded due to evidence of corrective lower limb surgery after baseline, which is smaller than the reported incidence of corrective surgery in children of up to 67%.^1^^,^^2^^,^^18^^,^^22^^,^^23^ This may also suggest some further benefit to prevent surgery, but longer-term follow-up of patients beginning burosumab at a young age are necessary to develop a clearer picture of the impact of burosumab on future surgery need.

Overall, this study has shown that switching from active D/Pi to burosumab significantly improves lower limb malalignment in children with XLH vs remaining on active D/Pi, with early age at burosumab initiation providing the greatest benefit, and with absence of evidence for shorter stature being disadvantageous (in contrast, shorter children had the greatest improvement in lower limb malalignment). Future analyses will expand upon these findings by assessing the durability of the response over the longer-term, the trajectory of continued improvement with extended treatment over the 10-yr DMP, the extent to which burosumab obviates the need for lower limb corrective surgery, and the specific factors associated with less potential for improvement including BMI, duration of burosumab exposure, precise age at initiation, whether an age or treatment duration threshold exists beyond which improvement is more or less likely, and the impact of lower limb malalignment type on long-term outcomes.

## Supplementary Material

XLH_DMP_LLM_Supplementary_Materials_REV_Legends_20May_CLEAN_zjaf079

XLH_DMP_LLM_Ward_CONSORT-_Consolidated_Standards_of_Reporting_Trials_zjaf079

## Data Availability

Data available upon reasonable request.

## References

[1] Ariceta G, Beck-Nielsen SS, Boot AM, et al. The international X-linked hypophosphatemia (XLH) registry: first interim analysis of baseline demographic, genetic and clinical data. Orphanet J Rare Dis. 2023;18(1):304. 37752558 10.1186/s13023-023-02882-4 PMC10523658

[2] Dahir K, Roberts MS, Krolczyk S, Simmons JH. X-linked hypophosphatemia: a new era in management. J Endocr Soc. 2020;4(12):bvaa151. 33204932 10.1210/jendso/bvaa151 PMC7649833

[3] Beck-Nielsen SS, Mughal Z, Haffner D, et al. FGF23 and its role in X-linked hypophosphatemia-related morbidity. Orphanet J Rare Dis. 2019;14(1):58. 30808384 10.1186/s13023-019-1014-8 PMC6390548

[4] Carpenter TO, Imel EA, Holm IA, Jan de Beur SM, Insogna KL. A clinician's guide to X-linked hypophosphatemia. J Bone Miner Res. 2011;26(7):1381–1388. 21538511 10.1002/jbmr.340 PMC3157040

[5] Al Juraibah F, Al Amiri E, Al Dubayee M, et al. Diagnosis and management of X-linked hypophosphatemia in children and adolescent in the Gulf cooperation council countries. Arch Osteoporos. 2021;16(1):52. 33660084 10.1007/s11657-021-00879-9 PMC7929956

[6] Bosman A, Appelman-Dijkstra NM, Boot AM, et al. Disease manifestations and complications in Dutch X-linked hypophosphatemia patients. Calcif Tissue Int. 2024;114(3):255–266. 38226986 10.1007/s00223-023-01172-2 PMC10901935

[7] Mindler GT, Kranzl A, Stauffer A, et al. Lower limb deformity and gait deviations among adolescents and adults with X-linked hypophosphatemia. Front Endocrinol (Lausanne). 2021;12:754084. 34646241 10.3389/fendo.2021.754084 PMC8503556

[8] Ikegawa K, Hasegawa Y. Presentation and diagnosis of Pediatric X-linked hypophosphatemia. Endocrines. 2023;4(1):128–137.

[9] Lambert AS, Zhukouskaya V, Rothenbuhler A, Linglart A. X-linked hypophosphatemia: management and treatment prospects. Joint Bone Spine. 2019;86(6):731–738. 30711691 10.1016/j.jbspin.2019.01.012

[10] Insogna KL, Rauch F, Kamenicky P, et al. Burosumab improved histomorphometric measures of osteomalacia in adults with X-linked hypophosphatemia: a phase 3, single-arm, international trial. J Bone Miner Res. 2019;34(12):2183–2191. 31369697 10.1002/jbmr.3843 PMC6916280

[11] Bonnet-Lebrun A, Linglart A, De Tienda M, et al. Quantitative analysis of lower limb and pelvic deformities in children with X-linked hypophosphatemic rickets. Orthop Traumatol Surg Res. 2023;109(3):103187. 34929395 10.1016/j.otsr.2021.103187

[12] Imel EA . Enthesopathy, osteoarthritis, and mobility in X-linked hypophosphatemia1. J Clin Endocrinol Metab. 2020;105(7):e2649. 10.1210/clinem/dgaa242 PMC7271761 32374835

[13] Imel EA, Glorieux FH, Whyte MP, et al. Burosumab versus conventional therapy in children with X-linked hypophosphataemia: a randomised, active-controlled, open-label, phase 3 trial. Lancet. 2019;393(10189):2416–2427. 31104833 10.1016/S0140-6736(19)30654-3 PMC7179969

[14] Linglart A, Imel EA, Whyte MP, et al. Sustained efficacy and safety of Burosumab, a monoclonal antibody to FGF23, in children with X-linked hypophosphatemia. J Clin Endocrinol Metab. 2022;107(3):813–824. 34636899 10.1210/clinem/dgab729 PMC8851952

[15] Makitie O, Doria A, Kooh SW, Cole WG, Daneman A, Sochett E. Early treatment improves growth and biochemical and radiographic outcome in X-linked hypophosphatemic rickets. J Clin Endocrinol Metab. 2003;88(8):3591–3597. 12915641 10.1210/jc.2003-030036

[16] Biosse Duplan M, Coyac BR, Bardet C, et al. Phosphate and vitamin D prevent periodontitis in X-linked hypophosphatemia. J Dent Res. 2017;96(4):388–395. 27821544 10.1177/0022034516677528

[17] Glorieux FH, Marie PJ, Pettifor JM, Delvin EE. Bone response to phosphate salts, ergocalciferol, and calcitriol in hypophosphatemic vitamin D-resistant rickets. N Engl J Med. 1980;303(18):1023–1031. 6252463 10.1056/NEJM198010303031802

[18] Haffner D, Emma F, Eastwood DM, et al. Clinical practice recommendations for the diagnosis and management of X-linked hypophosphataemia. Nat Rev Nephrol. 2019;15(7):435–455. 31068690 10.1038/s41581-019-0152-5 PMC7136170

[19] Linglart A, Biosse-Duplan M, Briot K, et al. Therapeutic management of hypophosphatemic rickets from infancy to adulthood. Endocr Connect. 2014;3(1):R13–R30. 24550322 10.1530/EC-13-0103 PMC3959730

[20] Portale AA, Ward L, Dahir K, et al. Nephrocalcinosis and kidney function in children and adults with X-linked hypophosphatemia: baseline results from a large longitudinal study. J Bone Miner Res. 2024;39(10):1493–1502. 39151033 10.1093/jbmr/zjae127 PMC11425691

[21] Santos F, Fuente R, Mejia N, Mantecon L, Gil-Pena H, Ordonez FA. Hypophosphatemia and growth. Pediatr Nephrol. 2013;28(4):595–603. 23179196 10.1007/s00467-012-2364-9

[22] Emma F, Cappa M, Antoniazzi F, et al. X-linked hypophosphatemic rickets: an Italian experts' opinion survey. Ital J Pediatr. 2019;45(1):67. 31151476 10.1186/s13052-019-0654-6 PMC6545008

[23] Higuchi C . Orthopedic complications and Management in Children with X-linked hypophosphatemia. Endocrines. 2022;3(3):488–497.

[24] Sharkey MS, Grunseich K, Carpenter TO. Contemporary medical and surgical management of X-linked hypophosphatemic rickets. J Am Acad Orthop Surg. 2015;23(7):433–442. 26040953 10.5435/JAAOS-D-14-00082

[25] Petje G, Meizer R, Radler C, Aigner N, Grill F. Deformity correction in children with hereditary hypophosphatemic rickets. Clin Orthop Relat Res. 2008;466(12):3078–3085. 18841431 10.1007/s11999-008-0547-2 PMC2628230

[26] Paludan CG, Thomsen KKV, Rahbek O, Kold S. Complications of orthopedic treatment in patients diagnosed with X-linked hypophosphatemic rickets. J Pediatr Endocrinol Metab. 2022;35(8):1003–1009. 35700440 10.1515/jpem-2021-0775

[27] Carpenter TO, Whyte MP, Imel EA, et al. Burosumab therapy in children with X-linked hypophosphatemia. N Engl J Med. 2018;378(21):1987–1998. 29791829 10.1056/NEJMoa1714641

[28] Ward LM, Hogler W, Glorieux FH, et al. Burosumab vs conventional therapy in children with X-linked hypophosphatemia: results of the open-label, phase 3 extension period. JBMR Plus. 2024;8(1):ziad001. 38690124 10.1093/jbmrpl/ziad001 PMC11059996

[29] Frumberg DB, Merritt JL, Chen A, Carpenter TO. Impact of burosumab on lower limb alignment in children with X-linked hypophosphatemia. J Pediatr Soc North Am. 2024;6:100012. 40433251 10.1016/j.jposna.2024.100012 PMC12088215

[30] Ward LM, Glorieux FH, Whyte MP, et al. Effect of burosumab compared with conventional therapy on younger vs older children with X-linked hypophosphatemia. J Clin Endocrinol Metab. 2022;107(8):e3241–e3253. 35533340 10.1210/clinem/dgac296 PMC9282253

[31] Whyte MP, Carpenter TO, Gottesman GS, et al. Efficacy and safety of burosumab in children aged 1-4 years with X-linked hypophosphataemia: a multicentre, open-label, phase 2 trial. Lancet Diabetes Endocrinol. 2019;7(3):189–199. 30638856 10.1016/S2213-8587(18)30338-3

[32] Sabharwal S, Zhao C. The hip-knee-ankle angle in children: reference values based on a full-length standing radiograph. J Bone Joint Surg Am. 2009;91(10):2461–2468. 19797583 10.2106/JBJS.I.00015

[33] Lassalle L, Regnard NE, Durteste M, et al. Evaluation of a deep learning software for automated measurements on full-leg standing radiographs. Knee Surg Relat Res. 2024;36(1):40. 39614404 10.1186/s43019-024-00246-1 PMC11606017

[34] Colantonio DA, Kyriakopoulou L, Chan MK, et al. Closing the gaps in pediatric laboratory reference intervals: a CALIPER database of 40 biochemical markers in a healthy and multiethnic population of children. Clin Chem. 2012;58(5):854–868. 22371482 10.1373/clinchem.2011.177741

[35] Quezada E, Lapidus J, Shaughnessy R, Chen Z, Silberbach M. Aortic dimensions in turner syndrome. Am J Med Genet A. 2015;167A(11):2527–2532. 26118429 10.1002/ajmg.a.37208

[36] Bout-Tabaku S, Shults J, Zemel BS, et al. Obesity is associated with greater valgus knee alignment in pubertal children, and higher body mass index is associated with greater variability in knee alignment in girls. J Rheumatol. 2015;42(1):126–133. 25362652 10.3899/jrheum.131349

[37] Walker JL, Hosseinzadeh P, White H, et al. Idiopathic genu valgum and its association with obesity in children and adolescents. J Pediatr Orthop. 2019;39(7):347–352. 31305377 10.1097/BPO.0000000000000971

[38] Schlegl AT, Szuper K, Somoskeoy S, Than P. Three dimensional radiological imaging of normal lower-limb alignment in children. Int Orthop. 2015;39(10):2073–2080. 26156714 10.1007/s00264-015-2851-2

[39] Akta C, Wenzel-Schwarz F, Stauffer A, et al. The ankle in XLH: reduced motion, power and quality of life. Front Endocrinol (Lausanne). 2023;14:1111104. 37033213 10.3389/fendo.2023.1111104 PMC10073673

[40] Mindler GT, Kranzl A, Stauffer A, Haeusler G, Ganger R, Raimann A. Disease-specific gait deviations in pediatric patients with X-linked hypophosphatemia. Gait Posture. 2020;81:78–84. 32688230 10.1016/j.gaitpost.2020.07.007

[41] Seefried L, Smyth M, Keen R, Harvengt P. Burden of disease associated with X-linked hypophosphataemia in adults: a systematic literature review. Osteoporos Int. 2020;32(1):7–22. 32710160 10.1007/s00198-020-05548-0 PMC7755619

[42] Skrinar A, Dvorak-Ewell M, Evins A, et al. The lifelong impact of X-linked hypophosphatemia: results from a burden of disease survey. J Endocr Soc. 2019;3(7):1321–1334. 31259293 10.1210/js.2018-00365 PMC6595532

[43] Scorcelletti M, Kara S, Zange J, et al. Lower limb bone geometry in adult individuals with X-linked hypophosphatemia: an observational study. Osteoporos Int. 2022;33(7):1601–1611. 35435480 10.1007/s00198-022-06385-z PMC9187561

