# Supplementary material for: Switching from active vitamin D and phosphate supplementation to burosumab significantly corrects lower limb malalignment in pediatric X-linked hypophosphatemia
Source: J Bone Miner Res. 2025 Jun 13;40(12):1332–42. doi: 10.1093/jbmr/zjaf079 (PMC12685721; doi:10.1093/jbmr/zjaf079)
Supplement: XLH_DMP_LLM_Supplementary_Materials_REV_Legends_20May_CLEAN_zjaf079 [file xlh_dmp_llm_supplementary_materials_rev_legends_20may_clean_zjaf079.docx]

XLH DMP Lower Limb Malalignment Manuscript

Supplementary Materials

**
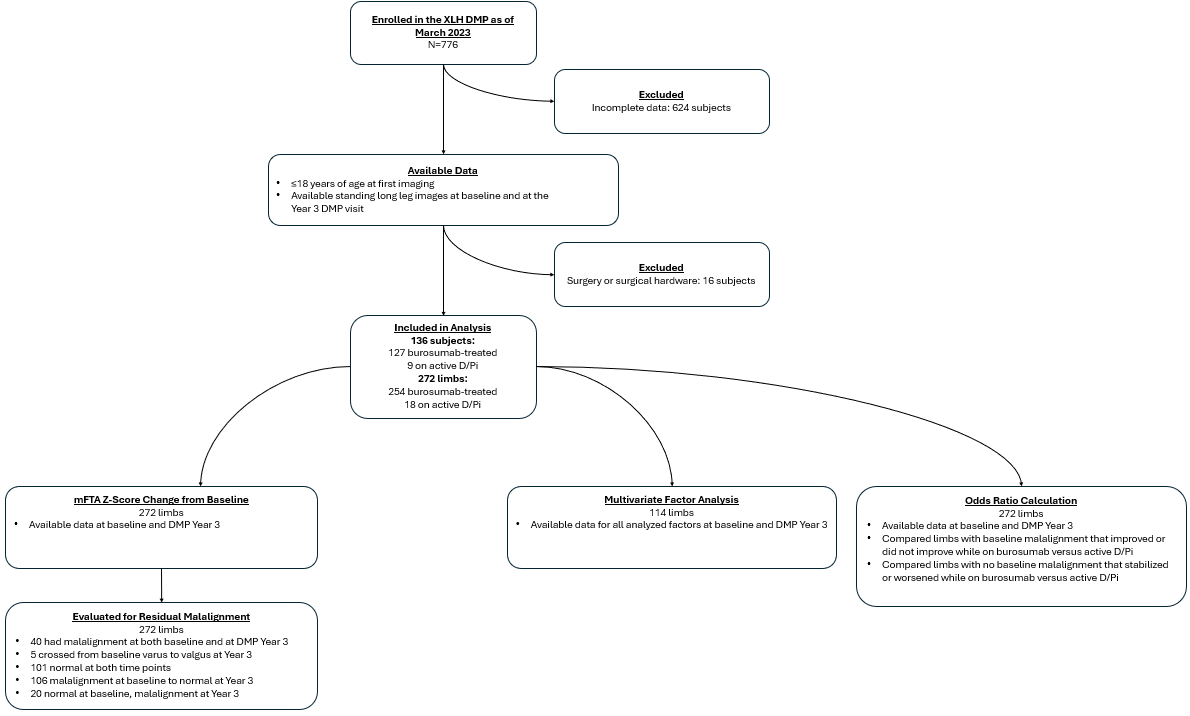
**

**Supplementary Figure: Patient Disposition Diagram.** Of the 776 patients enrolled in the XLH DMP as of March 2023, 136 were included in the present analysis (272 limbs). Patients were excluded due to incomplete data or confounding surgery or surgical hardware, as shown above. The 272 limbs were analyzed for change in mFTA Z-score from baseline at DMP Year 3 and evaluated for residual malalignment (272 limbs), analyzed for factors contributing to improvement in baseline malalignment (114 limbs), and underwent an odds ratio calculation to compare improvement, worsening, and stabilization (272 limbs).

**Supplementary Methods: Factors Assessed in Linear Mixed-Effects Model**

- 1. Treatment groups
  2. Age at enrollment in DMP
  3. Sex
  4. Age at diagnosis
  5. Age at initiation of treatment with conventional therapy (Phosphate)
  6. Age at initiation of treatment with conventional therapy (Active Vit D)
  7. Age at initiation of treatment with burosumab
  8. Height
  9. Weight
  10. Baseline height Z-score
  11. Change in height Z-score over the time of analysis
  12. Baseline body mass index
  13. Change in body mass index over the time of analysis
  14. Rickets Severity Score (Total) at baseline
  15. Rickets Severity Score (Knee) at baseline
  16. Serum phosphate Z-score at baseline
  17. Change in serum phosphate Z-score at Year 3
  18. Serum alkaline phosphatase at baseline
  19. Change in serum alkaline phosphatase at Year 3
  20. Serum intact parathyroid hormone at Baseline
  21. Burosumab dose (mg/kg)
  22. Serum 1,25-dihydroxyvitamin D
  23. Duration of burosumab exposure
  24. Positive *PHEX* variant

**Supplementary Table: Laboratory Values at Baseline and Final Visit by Treatment**

|  | **Active D/Pi to  Burosumab-Treated** | | **Active D/Pi Only*** | | **Total** | |
| --- | --- | --- | --- | --- | --- | --- |
|  | **Baseline** | **Final Visit** | **Baseline** | **Final Visit** | **Baseline** | **Final** |
| **Serum Phosphate, mmol/L; mean (SD)** | 1.0 (0.2) | 1.1 (0.2) | 0.9 (0.1) | 0.8 (0.2) | 1.0 (0.2) | 1.1 (0.2) |
| **Serum Phosphate Z-score; mean (SD)** | -2.6 (1.0) | -1.7 (0.7) | -3.2 (0.7) | -3.4 (0.9) | -2.6 (1.0) | -1.8 (0.8) |
| **Serum Alkaline Phosphatase, U/L; mean (SD)** | 418.8 (140.0) | 291.9 (124.1) | 446.6 (161.1) | 525.8 (140.7) | 420.4 (140.8) | 302.1 (133.0) |
| **Serum Alkaline Phosphatase Z-score; mean (SD)** | 2.4 (1.9) | 0.9 (1.5) | 2.8 (2.6) | 4.0 (2.7) | 2.4 (2.0) | 1.1 (1.6) |
| **Serum iPTH, pg/mL; mean (SD)** | 47.3 (27.4) | 50.1 (21.0) | 53.5 (36.5) | 129.4 (162.1) | 47.7 (28.0) | 54.0 (42.7) |
| **Serum 1,25 Vitamin D, pg/mL; mean (SD)** | 53.6 (18.8) | 63.8 (15.8) | 46.8 (15.8) | 41.1 (16.0) | 53.2 (18.6) | 62.5 (16.6) |

**Supplementary Table: Laboratory Values at Baseline and Final Visit by Treatment.** Mean (SD) laboratory values at baseline and last visit are reported for patients by treatment group (those who switched to burosumab and those who remained on active D/Pi), and summed.
